# Construction of Synthetic Microbial Community with Core Microorganisms for Soy Sauce Fermentation

**DOI:** 10.3390/foods15101736

**Published:** 2026-05-14

**Authors:** Pengfei Chen, Guocheng Du, Jian Chen, Fang Fang

**Affiliations:** 1 Science Center for Future Foods, Jiangnan University, Wuxi 214122, China; 6230210048@stu.jiangnan.edu.cn (P.C.); gcdu@jiangnan.edu.cn (G.D.); jchen@jiangnan.edu.cn (J.C.); 2 School of Biotechnology and Key Laboratory of Industrial Biotechnology of Ministry of Education, Jiangnan University, Wuxi 214122, China; 3 State Key Laboratory of Food Science and Resources, Jiangnan University, Wuxi 214122, China; 4 Key Laboratory of Carbohydrate Chemistry and Biotechnology, Ministry of Education, Jiangnan University, Wuxi 214122, China; 5 National Engineering Research Center of Cereal Fermentation and Food Biomanufacturing, Jiangnan University, Wuxi 214122, China

**Keywords:** soy sauce, core microbes, synthetic microbial community, metabolomics, metagenomics

## Abstract

Core microbes and succession of the microbial community greatly influence soy sauce fermentation process. This study identified seven functionally important core microbes, including *Weissella paramesenteroides, Lactiplantibacillus plantarum, Tetragenococcus halophilus, Pediococcus pentosaceus, Zygosaccharomyces rouxii, Candida orthopsilosis*, and *Aspergillus oryzae* for soy sauce fermentation, based on dominant taxa, co-occurrence relationships, and volatile-associated taxa analysis. Four distinct fermentation phases were identified for soy sauce fermentation based on metagenomics and metabolomics data correlation analyses. Acceptable fermentation performance and comparable soy sauce flavor compounds were achieved using a temporal synthetic microbial community for fermentation. The synthetic microbial community was assembled with inoculation of dominant lactic acid bacteria (LAB) in the immediate early phase, other LAB in early and middle phases, and yeasts in the late phase. Glutamate and 4-ethylguaiacol were identified as soy sauce fermentation indicators for early to middle and late fermentation phases, respectively. These results may provide a possible solution for achieving precise control over the brewing process and improving the flavor and quality of soy sauce.

## 1. Introduction

Soy sauce, a traditional fermented condiment, is produced through the synergistic actions of microorganisms, including molds, bacteria, and yeasts [1]. These microorganisms decompose macromolecules such as proteins and starch found in soybeans and wheat, generating essential components such as volatile flavor compounds, amino acids, and organic acids that contribute to the unique taste and nutritional characteristics of soy sauce [2,3]. Recent advances in high-throughput sequencing technologies have elucidated the complexity and dynamics of microbial communities during soy sauce fermentation. Alongside the heterogeneity in fermentation system scale and environmental variability, soy sauce fermentation process can be affected by three aspects: microbial diversity and succession patterns [4], the influence of spatial heterogeneity [5], and the regulatory role of environmental factors [6]. Soy sauce production is a half-open fermentation process; the microbial community is easily contaminated with non-functional microorganisms and environmental microbes through natural inoculation, leading to instability in aroma and quality [6]. Furthermore, procedures for soy sauce production vary in different regions, evaluation of fermentation status often depends on empirical judgment. Core microbes comprising dominant and functional microorganisms are capable of driving soy sauce fermentation process [7]. Thus, systematic construction of synthetic microbial consortia for soy sauce fermentation may have the superiority in robust production of soy sauce.

Soy sauce fermentation is a complex, multi-stage process that involves a diverse array of microbial taxa, including bacteria such as *Staphylococcus, Bacillus, Weissella, Lactococcus,* and *Tetragenococcus*, as well as fungi such as *Aspergillus* and *Zygosaccharomyces*. These microorganisms collectively contribute to nutrient hydrolysis and flavor development [8,9,10,11]. Recognition of essential microbes and characterization of their functions and metabolic profiles are critical for construction of artificial microbial community for soy sauce fermentation. In previous studies, six microorganisms, including *Aspergillus oryzae, Wickerhamomyces anomalus, Zygosaccharomyces rouxii, Staphylococcus carnosus, Weissella paramesenteroides,* and *Tetragenococcus halophilus,* were identified as the core microorganisms for soy sauce fermentation [12]. Another three bacterial genera, *Enterococcus, Bacillus, and Pediococcus,* were also found to be closely associated with soy sauce quality [5]. A preliminary synthetic microbial community comprising six microbial genera was used in a salt-reduced soy sauce fermentation process, which demonstrated the potential applications of synthetic consortia in food fermentation and microbiome engineering [12]. Both top-down and bottom-up approaches were used to develop a synthetic microbial community for brewing Huangjiu (Chinese rice wine). Employment of the synthetic microbial community not only improved fermentation efficiency but also maintained the unique flavor characteristics of Huangjiu [13]. However, some selected microbial communities may include functionally redundant or non-contributing microorganisms. Identification and selection of core microorganisms for construction of synthetic microbial community can be optimized. Moreover, the structure of synthetic microbial consortia is susceptible to alterations during continuous fermentation, due to the extended fermentation process of soy sauce production. Therefore, synthetic microbial communities cannot be assembled through single inoculation and must be meticulously designed and assembled with consideration of the optimal functioning conditions for each member. We hypothesize that temporal synthetic microbial community (SynCom) improves functional output vs. static consortia. Moreover, indicators apart from amino acid nitrogen and total acidity that can reflect the role of synthetic microbial community, as well as precisely evaluate soy sauce fermentation process, are required.

This study aims to identify and validate core microorganisms involved in soy sauce fermentation by correlating microbial community dynamics with formation of flavor compounds during soy sauce fermentation. Principles for assembling a practical SynCom with core microbes will be investigated, and the quality and aroma of soy sauce produced with the SynCom will be evaluated. Results of this work may provide potential solutions for optimizing soy sauce fermentation, thereby enhancing both process stability and product quality.

## 2. Materials and Methods

### 2.1. Strains and Cultivation

*Z*. *rouxii* (ZQ-01), *A*. *oryzae* (3.042), *T*. *halophilus* (R44), *Staphylococcus epidermidis* (HC-1), and *Lactiplantibacillus plantarum* (JL-20) used in this study are obtained from the lab stocks. *Candida orthopsilosis* (CV-13), *Pediococcus acidilactici* (PP-7), *Pediococcus pentosaceus* (PP-11), and *W*. *paramesenteroides* (WP-8) were isolated from soy sauce moromi by plating of severely diluted soy sauce moromi on isolating plates in this work. YPD and MRS media were used for isolating *C. orthopsilosis* and lactic acid bacteria (LAB), respectively. Bacterial and yeast strains were identified by blasting sequences of the amplicons of genomic DNAs amplified with primers 27F and 1492R and primers NS1 and NS8 [14] against NCBI, respectively. *A. oryzae* was cultivated in PDA at 30 °C, *Z. rouxii* and *C. orthopsilosis* were cultivated in YPD medium at 30 °C. LAB were cultivated in MRS broth supplemented with 5–10% (*w*/*v*) NaCl at 30 °C or 37 °C. *S. epidermidis* was grown in nutrient broth and cultivated at 37 °C. For determination of viable cells of microorganisms, serial dilutions of soy sauce moromi were plated on the appropriate agar (LB for non-LAB bacteria, MRS for LAB, and PDA for fungi) plates.

### 2.2. Materials and Reagents

Defatted soybeans, flour, and salt used in this study were all purchased from local market in Wuxi, Jiangsu province, China. Methanol and acetonium were obtained from Merck Ltd. (Shanghai, China), and sulfuric acid was purchased from Sinopharm Chemical Reagent Co., Ltd. (Shanghai, China). Derivatization reagent o-phthaldialdehyde (OPA) was purchased from Agilent Technologies Co., Ltd. (Santa Clara, CA, USA), amino acid standard mixture was purchased from Sigma-Aldrich Co., Ltd. (Saint Louis, MO, USA), and organic acid standards were purchased from Sigma-Aldrich Co., Ltd. (Saint Louis, MO, USA). All other chemicals of analytical purity were purchased from Sinopharm Chemical Reagent Co. (Shanghai, China).

### 2.3. Soy Sauce Fermentation

Traditional soy sauce fermentation was conducted for 40 days. Soybean meal is soaked in 1.1 times of water (*v*/*w*) for 2 h, then sterilized at 121 °C (0.1 MPa) for 20 min. After cooling to room temperature, soybean meal was thoroughly mixed with wheat flour at a ratio of 7:3 (*w*/*w*), and then inoculated with 0.2% (*w*/*w*) of *A*. *oryzae* 3.042 (1 × 10^7^ spores/g) before cultivating at 30 °C and 90% relative humidity for 42–46 h. One kilogram of the mature koji was combined with 2 L of brine to make a moromi mash containing 18% (*w*/*w*) salt. The mash was transferred to a 5 L beaker covered with a gauze layer. Soy sauce fermentation commenced at 16 °C, then the temperature rose by 2 °C daily until it reached 30 °C. *Z. rouxii* ZQ01 (1.0 × 10^7^ CFU/g) and *C. orthopsilosis* (1.0 × 10^7^ CFU/g) were added on day 25–30 of fermentation (when pH = 5.3). Moromi samples were taken at different time intervals and were kept at −80 °C. Every setup of soy sauce fermentation was conducted in three independent batches.

For soy sauce fermentation using synthetic microbial community (SynCom) and the corresponding control, a modified koji-making procedure was employed. Briefly, in 0–24 h, koji was prepared in a sterile beaker at 30 °C and 90% relative humidity. Then the koji was crushed and transferred to a sterile tray and incubated under the same conditions. The koji was turned over every 6 h until the mycelia predominantly turned yellow, resulting in the mature koji. Preparation of soy sauce moromi mash was the same as traditional soy sauce fermentation. Fermentation was performed at 30 °C for 40 d. In order to maximize the representation of the function of each strain and to reduce the impact of the quantitative differences in fermentation validation, we assumed that each core microorganism has the same ecological niche. *W. paramesenteroides, L. plantarum, T. halophilus, P. pentosaceus, P. acidilactici,* and *S. epidermidis* were inoculated at the assigned time (0 d, 5 d, or 10 d) with the same concentration (1.0 × 10^8^ CFU/g). *Z. rouxii* and *C. orthopsilosis* were added on 25 days after initiation of brine fermentation. For each group, fermentation is conducted in three identical containers in parallel. For strain omission soy sauce fermentation, moromi inoculated with all tested strains was used as the control group. A traditional soy sauce fermentation was used as the control group for SynComs fermentation.

### 2.4. Determination of Physicochemical Properties of Soy Sauce

Soy sauce moromi samples were diluted and vortexed for 10 min before proceeding with analysis. pH was determined with a pH meter (FE20K, Mettler Toledo, Columbus, OH, USA). Amino acid nitrogen, total acid, and total nitrogen content were analyzed as described in previous work [15]. Reducing sugars were quantified using the DNS method [15]. 1 mL sample was mixed with 1 mL DNS reagent and incubated in boiling water for 5 min before measuring the absorbance at 540 nm. Glucose was used to construct standard curve. Protein relative utilization rate was calculated as: Protein relative utilization rate (%) = (AA/TN) × 100%, where AA is amino acid content, and TN is total nitrogen content [16]. Non-volatile metabolites, including organic acids and free amino acids, were quantified using high-performance liquid chromatography (HPLC, Agilent Technologies Co., Ltd., Santa Clara, CA, USA). For organic acids analysis [5], an Aminex HPX-87H column (9 μm, 7.8 mm × 300 mm, Thermo Fisher Scientific, Waltham, MA, USA) was used. Separation was carried out at a column temperature of 40 °C, using 5 mmol/L H_2_SO_4_ as the eluent with a flow rate of 0.5 mL/min. UV detection was set to 210 nm. For free amino acids analysis [17], an Agilent ODS Hypersil column (5 μm, 4.6 mm × 250 mm, Thermo Fisher Scientific, Waltham, MA, USA) was employed. Separation was carried out at a column temperature of 40 °C, employing mobile phase A comprising 5 g/L sodium acetate (containing 0.2 mL/L triethylamine and 5 mL/L tetrahydrofuran) and mobile phase B composed of 5 g/L sodium acetate, methanol, and acetonitrile in a 1:2:2 (*v*/*v*) ratio, at a flow rate of 1.0 mL/min, with UV detection set to 338 nm.

### 2.5. High-Throughput Sequencing

Total genomic DNA of the soy sauce moromi samples was isolated with OMEGA E.Z.N.A.™ Mag-Bind Soil DNA Kit (Omega Bio-Tek, Norcross, GA, USA) according to the manufacturer’s instructions. For bacterial community analysis, the V3-V4 region of the 16S rRNA was amplified with primers 341F (5′-CCTACGGGNGGCWGCAG-3′) and 805R (5′-GACTACHVGGGTATCTAATCC-3′). For fungal community analysis, the ITS1-ITS2 region of ITS was targeted using primers ITS1F (5′-CTTGGTCATTTAGAGGAAGTAA-3′) and ITS2R (5′-GCTGCGTTCTTCATCGATGC-3′). The obtained sequences were then used to construct libraries on an Illumina HiSeq™ platform. Sequencing data were analyzed by Sangon Biotech Co., Ltd. (Shanghai, China). Bacterial ASVs were taxonomically annotated against the SILVA database (version 138), while fungal ASVs were annotated using the UNITE database. Analysis of alpha and beta diversity was performed with QIIME [18].

### 2.6. Untargeted Metabolomics Analysis

Untargeted metabolomics analysis of moromi samples was performed using a UPLC-ESI-MS/MS system (UPLC, Thermo Scientific Vanquish, Waltham, MA, USA; MS, Applied Biosystems QExactive HF-X, Waltham, MA, USA) by Sangon Biotech (Shanghai) Co., Ltd. [17]. For QC analysis, an extract of ten samples was mixed and used to assess the reproducibility of the analytical process. Separation of one aliquot in positive and negative ion mode was performed on a T3 column (Waters ACQUITY Premier HSS T3, 1.8 µm, 2.1 mm × 100 mm, Waters Technology Co., Ltd., Milford, MA, USA) maintained at 40 °C. Gradient elution was carried out using solvent A (0.1% formic acid in water) and solvent B (0.1% formic acid in acetonitrile) at a flow rate 0.4 mL/min with the following program: 5–20% B in 1 min, 20–99% B in 2 min (hold 1.5 min), then re-equilibrated to 5% B in 0.1 min and held for 1.4 min. The injection volume was 4 µL. Raw mass spectrometry data were converted to mzML format using ProteoWizard, and peak extraction, alignment, and retention time corrections were performed using the XCMS software (Version 2025). Peaks with a missing value rate >50% in each sample group were filtered out. For blank values >50%, the 1/5 minimum value method was used for imputation, while for blank values <50%, the KNN method was used. The corrected and filtered peaks were identified by searching the laboratory’s in-house database, integrating public databases, and utilizing prediction libraries. Finally, compounds with a combined identification score of 0.5 or higher and a CV value of less than 0.3 in QC samples were extracted, and the positive and negative modes were merged.

Volatiles were analyzed using headspace solid-phase microextraction (HS-SPME) coupled with gas chromatography (Agilent 7890 GC system, Agilent Technologies Co., Ltd., Santa Clara, CA, USA) and mass spectrometry (LECO Pegasus BT MS system, LECO Corporation, Saint Joseph, MI, USA), and equipped with a DB-WAXA column (30 m × 0.25 mm × 0.25 μm, Thermo Fisher Scientific, Waltham, MA, USA), as described previously [19]. 2-octanol (50 μg/L) was used as an internal standard; identification of compounds was achieved by comparing their mass spectra and retention indices (RIs) with those of authentic standards [20]. The parameters for analysis were set as follows: the extraction fiber was aged at 250 °C for 3 min. After incubating the sample at 60 °C for 2 min, extraction occurs for 25 min, followed by desorption at 250 °C for 60 s at the injection port. The GC method utilized a steady carrier gas flow of 1 mL/min. The temperature program: began at 40 °C (isothermal for 3 min), then increased by 10 °C per minute to 230 °C, where it was held for an additional 6 min. The interface was maintained at 250 °C. MS conditions: the electron impact ion source operating at 70 eV and 210 °C. Data were acquired within a mass range of 33–440 Da. Results were filtered and identified based on signal-to-noise ratios exceeding 30, match similarity scores above 700, and retention indices. The quantification of flavor volatiles was calculated using the internal standard method. content of flavor compounds (μg/L) = Peak area of the compound × Internal standard concentration/Peak area of the internal standard. The standard curve of each key compound was plotted by preparing gradient samples of 10 μg/L to 100 μg/L and analyzed under the same chromatographic conditions (Table S1).

### 2.7. Sensory Evaluation

The sensory evaluation panel consisted of eight professional members who conducted a sensory evaluation of soy sauce in accordance with GB/T 18186-2000 [21] to describe the flavor and aroma of the soy sauce samples. Panel members evaluated the intensity of each attribute using a linear scale ranging from 0 (none) on the left to 9 (very strong) on the right.

### 2.8. Statistical and Data Analysis

All results are expressed as the mean ± standard error (SE) from three independent biological experiments. Statistical analysis was conducted using IBM SPSS Statistics (Version 26.0, IBM Corp., Armonk, NY, USA). One-way analysis of variance (ANOVA) with Tukey’s post hoc test was employed to determine statistical significance among samples. Two-way ANOVA was used to determine statistical significance of changes among samples, which was set at *p* < 0.05. Point-line graphs were plotted using OriginPro (Version 2024, OriginLab Corp., Hampton, MA, USA). Cluster analysis and principal component analysis (PCA) were implemented using the factoextra package (version 1.0.7) from RStudio, and pairwise Spearman correlations were calculated using the corr. test from RStudio (Version 3.5.1). Correlations exhibiting high significance (*p* < 0.01) and high strength (|R| > 0.8) were visualized using Cytoscape (v.3.9.1). Co-occurrence networks were constructed based on Spearman correlations. *p*-values were corrected for multiple testing using FDR (Benjamini–Hochberg, q < 0.05). Only significant correlations with |r| > 0.6 were used as edges. The OmicShare online platform was used for generating cluster heatmaps, principal component analysis (PCA), correlation heatmaps, and network graphs. Stacked bar plots were generated via the online Chiplot (https://www.chiplot.online/).

## 3. Results and Discussion

### 3.1. Identification of Core Microbes During Soy Sauce Fermentation

To preliminarily identify the key microorganisms involved in soy sauce fermentation process, the criteria for selection of microbes was defined as, including dominant microorganisms (relative abundance > 1% and are present in ≥ 80% of samples), hub microorganisms (node connectivity threshold greater than 9 links in the co-occurrence network, q < 0.05), and flavor-contributing microorganisms (significant correlation (|r| > 0.5, *p* < 0.05, q < 0.05) with at least five volatile compounds).

**Figure 1:**
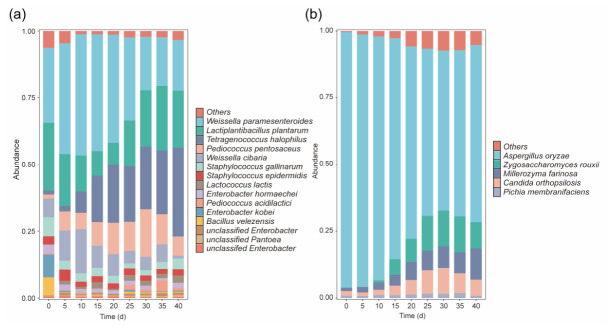
Successions of microbial composition in the process of soy sauce fermentation: (**a**), bacteria; (**b**), fungi.

A total of 55 bacterial and 28 fungal genera were identified in moromi samples taken from traditional soy sauce fermentation process (Table S2). As shown in Figure 1, in early fermentation phase (0–5 d), *Weissella* and *Lactiplantibacillus* were the dominant bacterial genera (mean relative abundance ≥ 10%), while *Pediococcus*, *Staphylococcus, Enterobacter*, and *Bacillus* were sub-dominant genera (1% ≤ mean relative abundance < 10%). In the middle to late fermentation phase (5–40 d), *Pediococcus* and *Tetragenococcus* gradually became the dominant genera, whereas the relative abundances of *Weissella* and *Lactobacillus* progressively decreased. From day 0 to day 15, *Aspergillus* was the dominant fungus in early to middle fermentation phase (0–15 d), while *Candida, Zygosaccharomyces,* and *Millerozyma* were the dominant fungi in middle to late fermentation phase (20–40 d). According to the top 1% relative abundance criteria, twelve bacterial species (*W*. *paramesenteroides*, *L*. *plantarum, Lactococcus lactis, T*. *halophilus, P*. *pentosaceus, P*. *acidilactici, S*. *epidermidis, Staphylococcus gallinarum, Bacillus velezensis, Weissella cibaria, Enterobacter hormaechei*, and *Enterobacter kobei*) and five fungi (*Z*. *rouxii, C*. *orthopsilosis, Millerozyma farinosa, A*. *oryzae, and Pichia membranifaciens*) were identified as the high-abundance fermentative microbes.

**Figure 2:**
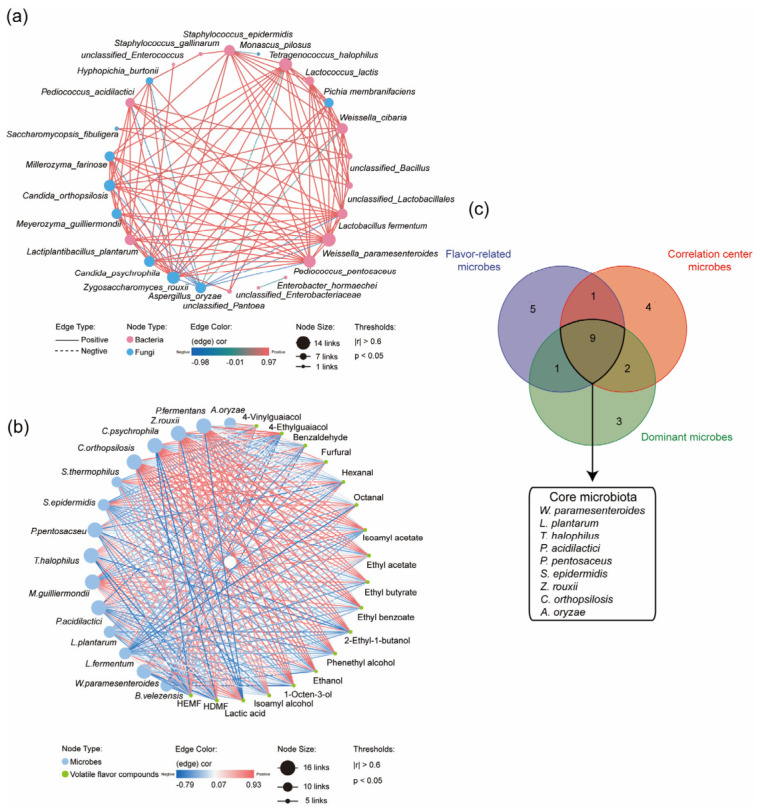
Identification of core microorganisms involved in soy sauce fermentation: (**a**), correlation network of cooccurring genera in the process of soy sauce fermentation (*p* < 0.05); (**b**), correlation networks between microbial genera and volatiles (*p* < 0.05); (**c**), deduction of core microorganisms for soy sauce fermentation.

A microbial co-occurrence network comprising 21 nodes and 204 edges was established based on Spearman correlations between microbial relative abundances (Figure 2a and Table S3). Potential core microorganisms were subsequently identified based on a node connectivity threshold of more than 9 links [22]. The identified hub microorganisms included nine bacteria: *W. paramesenteroides, L. plantarum, L. fermentum, L. lactis, T. halophilus, P. pentosaceus, P. acidilactici, S. epidermidis, and W. cibaria,* and seven *fungi: Z. rouxii, P. membranifaciens, C. orthopsilosis, M. guilliermondii, A. oryzae, M. farinosa, and C. psychrophile* (Figure 2a). To characterize functional microorganisms, co-occurrence analysis was conducted to explore the relationship between volatiles and dynamic microbial compositions. A total of 128 volatiles were detected in soy sauce, including 16 alcohols, 6 phenols, 10 pyrazines, 21 aldehydes, 26 ketones, 47 esters, and 9 acids (Table S4). As illustrated in Figure 2b, nine bacterial species (*W*. *paramesenteroides*, *L*. *plantarum*, *L*. *fermentum*, *Saccharopolyspora thermophila*, *T*. *halophilus*, *P*. *pentosaceus*, *P*. *acidilactici*, *S*. *epidermidis*, *Bacillus velezensis*) and six fungal species (*Z*. *rouxii, Pichia fermentans, C*. *orthopsilosis, Meyerozyma guilliermondii, A*. *oryzae, Candida guilliermondii*) were significantly correlated with 18 specific volatiles in soy sauce.

The final set of core microbes in soy sauce moromi in this work was comprehensively identified as *W. paramesenteroides*, *L. plantarum, T. halophilus, P. pentosaceus, P. acidilactici, S. epidermidis, A. oryzae, Z. rouxii*, and *C. orthopsilosis*, based on their relative abundance, frequent distribution, significant contributions to flavor formation, and pivotal roles within the microbial correlation networks (Figure 2c). *W. paramesenteroides, L. plantarum, T. halophilus, P. pentosaceus, P. acidilactici*, and *S. epidermidis* have frequently been identified as core or functional microorganisms in soy sauce fermentation in previous studies [23,24]. *A. oryzae* is the essential microorganism for making soy sauce koji [25]. *Z. rouxii* and *C. orthopsilosis* are typically identified as functional microorganisms due to their association with flavor volatiles [26]. The methods for identifying core microorganisms described above are primarily based on correlation analysis and have limitations when it comes to elucidating the relationship between microorganisms and food fermentation. Thus, metatranscriptomic or metabolomic analysis of relationships between microorganisms and their fermentation functions, as well as microbial fermentation experiments, are necessary for validation of their roles in soy sauce fermentation.

### 3.2. Validation of Core Microorganisms for Construction of Synthetic Microbial Community

Previous studies have identified core microorganisms for soy sauce fermentation based on correlation analysis rather than direct functional verification [27,28]. The actual roles of these core microorganisms can be more accurately evaluated by constructing synthetic microbial community containing these core microorganisms for soy sauce fermentation. Our objectives were to identify the minimal microbial composition for soy sauce fermentation and distinguish microorganisms with similar functional roles. Thus, composition of core microbes for soy sauce fermentation was systematically evaluated by omitting individual members and comparing the fermentation outcomes with those of the intact consortium. For soy sauce, the distinct phenolic aroma of 4-ethylguaiacol (4-EG) is generally regarded as a marker of good aroma [29]. Low level or absence of 4-EG in soy sauce leads to a loss of the signature “mellowness” in aroma [30]. A high level of amino acid content is related to high quality of soy sauce. Umami amino acids constitute 30–40% of the free amino acids in soy sauce, serving as the primary contributors to the umami taste (Figure S1). Both of them have been independently linked to consumer preference in prior literature. Thus, umami amino acids and 4-EG were primarily selected as indicators for evaluation of soy sauce fermentation process in this work.

**Figure 3:**
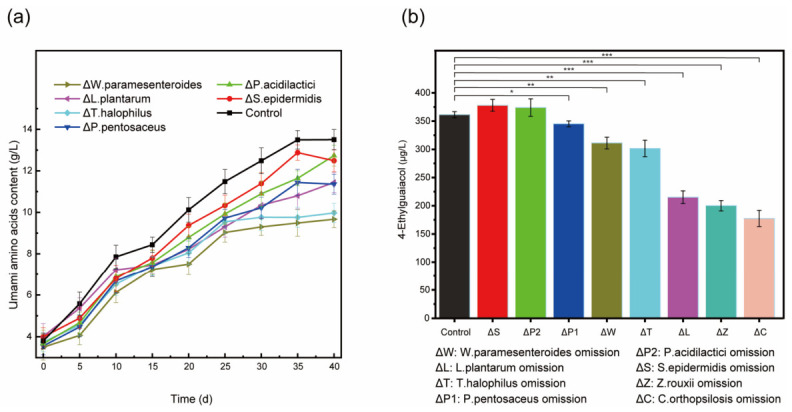
Conduction of soy sauce fermentation with core microorganisms: Changes in (**a**) umami amino acids and (**b**) 4-EG contents during soy sauce fermentation. *, 0.01 ≤ *p* < 0.05; **, 0.005 ≤ *p* < 0.01; ***, *p* < 0.005. Significance symbols indicate comparisons between each experimental group and the control group.

As shown in Figure 3a, omissions of *P. acidilactici* or *S. epidermidis* had slight influences on umami amino acids content, while omissions of *W*. *paramesenteroides, P*. *pentosaceus, T*. *halophilus,* or *L*. *plantarum* significantly decreased umami amino acids content by 27.2%, 15.8%, 25.9% and 15.5% compared to the control group, respectively. Omissions of microorganisms other than *P. acidilactici* or *S*. *epidermidis* significantly reduced production of 4-EG, which was decreased by 42.8%, 45.6%, and 56.5% with the omissions of *L. plantarum, Z*. *rouxii, or C*. *orthopsilosis* compared to the control group (Figure 3b). Integrating both soy sauce fermentation indicators, the core microbes for soy sauce fermentation were identified as seven microbial species: *W. paramesenteroides, L. plantarum, T. halophilus, P. pentosaceus, A. oryzae, Z. rouxii*, and *C. orthopsilosis*.

The identification of highly expressed genes and functional genes, coupled with the analysis of microbial interactions, is commonly utilized to narrow down the diversity of microorganisms of interest in Baijiu fermentation, thereby optimizing the artificial microbial community [31]. Several studies have employed machine learning techniques to elucidate the relationship between key flavor compounds in Baijiu and the predominant microorganisms. These approaches facilitate the auxiliary prediction of core microbial populations, thereby achieving microbial streamlining [32]. The omission experiment employed for shaping core microbes in this work cannot fully exclude the possibility that quantitative changes in volatile compounds are influenced by both inoculum size and shifts in total metabolic activity. Therefore, the omission experiment results do not directly represent the true relationship between the omitted species and the specific fermentation function. Future experiments using a constant total inoculum (e.g., by supplementing with inert biomass or a non-functional control strain) or performing reassembly experiments with adjusted cell ratios would help disentangle biomass effects from species-specific functions. In addition, consideration of the interactions and concentration ratios of the microorganisms in correlation with the natural fermentation ecology could be used for developing more precise criteria.

### 3.3. Characterization of Principles for Assembly of Synthetic Microbial Community

It was noted that umami amino acid levels in all strain-omitted experimental groups were lower than those in the control group (Figure 3a). This indicated that the synthetic microbial community used for soy sauce fermentation is not a simple combination of microbial strains, necessitating the identification of principles for microbial community construction. Therefore, principles such as precise indicators for monitoring of fermentation, division of fermentation phases, and combination of microorganisms were investigated to find suitable conditions for construction of synthetic microbial community.

**Figure 4:**
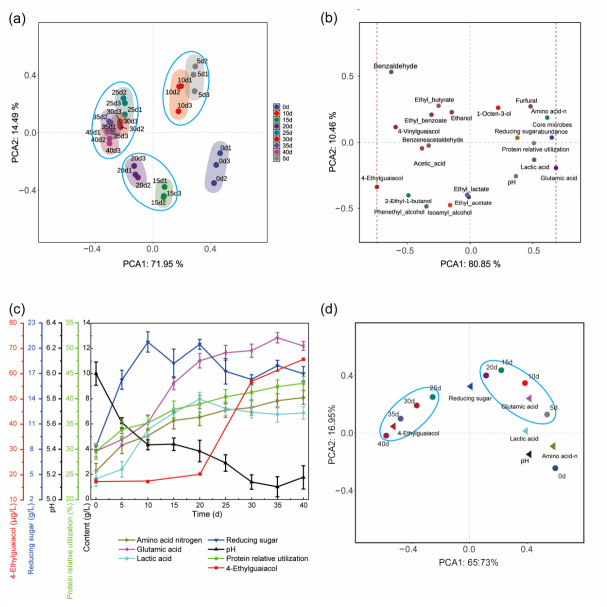
Characterization of indicators for monitoring soy sauce fermentation: (**a**) Principal component analysis of fermentation days with microbial community (*p* < 0.01); (**b**) Principal component analysis of indicators and metabolites in the process of soy sauce fermentation (*p* < 0.01); (**c**) Dynamic changes of candidate indicators in the process of soy sauce fermentation; (**d**) Principal component analysis of indicators over fermentation days.

Principal component analysis (PCA) was conducted to evaluate changes in volatiles, biological indicators, and abiotic indicators at different fermentation times. As shown in Figure 4a, structures of microbial community in the process of soy sauce fermentation were distinguished into four parts. Among the analyzed indicators, those situated closer to the right on PC1 exert a greater influence during the early fermentation phase [33]. Indicators closely associated with this phase included glutamic acid, core microorganisms abundances, amino nitrogen, relative protein utilization, lactic acid, and reducing sugars (Figure 4b). In contrast, indicators positioned closer to the left on PC1 have more significant impact on the late fermentation phase [33]. Thus, 4-ethylguaiacol is the indicator closely related to late fermentation phase (Figure 4b). Subsequently, changes in these candidate indicators throughout the entire fermentation process were examined (Figure 4c). Amino acid nitrogen, glutamic acid, lactic acid, reducing sugars, and pH exhibited pronounced variations in early to middle fermentation phases, while 4-EG showed marked changes in late fermentation phase. Content of reducing sugars increased to 21.1 g/L on day 10, fluctuated between 18 and 20 g/L from days 10 to 20, and then decreased. It showed no correspondence to fermentation patterns. Since pH is not an intuitive indicator, changes in lactic acid can serve as a reliable reference. As shown in Figure 4d, glutamic acid correlated closely with the early-to-middle fermentation phase (5–20 d), while 4-EG correlated strongly with the late fermentation phase (25 d-end of fermentation), based on the principal component analysis of these indicators over fermentation time. Therefore, glutamic acid was employed as the indicator in early fermentation phase, while 4-EG (4-ethylguaiacol) was used as the indicator in the late fermentation phase for evaluation of both soy sauce quality and flavor.

**Figure 5:**
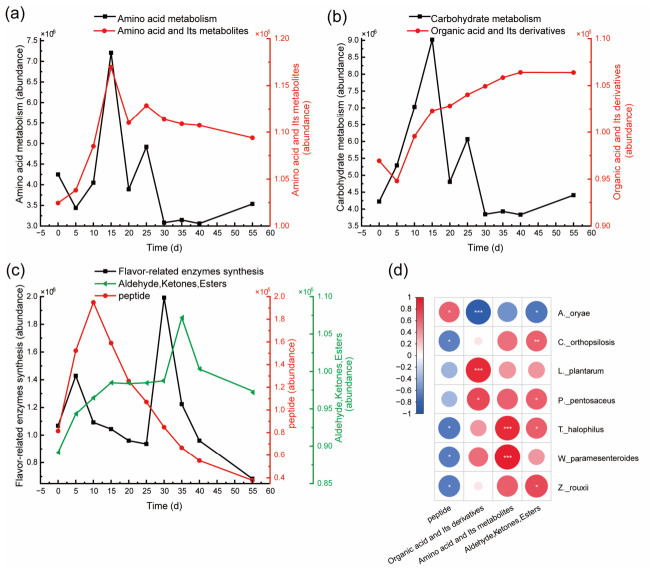
Dynamic changes of main metabolites in the process of soy sauce fermentation: (**a**), amino acids metabolism; (**b**), carbohydrates metabolism; (**c**), flavor-related enzymes synthesis; (**d**), correlations between core microorganisms and metabolites. *, 0.01 ≤ *p* < 0.05; **, 0.005 ≤ *p* < 0.01; ***, *p* < 0.005.

In order to characterize active periods for each core microorganism, the relationship between metabolic functions, metabolites, and soy sauce fermentation stages was investigated. The results showed that amino acids metabolisms were active in immediate early fermentation phase (0–5 d), early fermentation phase (5–15 d), and middle fermentation phase (15–25 d) (Figure 5a), while organic acids metabolisms were predominantly active in early and middle fermentation phases (Figure 5b). Flavor-related enzymes synthesis for formation of peptides and aldehydes, ketones, or esters was active in immediate early to early fermentation phase (0–10 d), and late fermentation phase (25–40 d), respectively (Figure 5c). These findings suggested differences in microbial metabolisms in distinct fermentation phases. Correspondence analysis between core microorganisms and metabolic phases allows for the segmentation of the soy sauce fermentation procedure and the identification of corresponding microorganisms. As shown in Figure 5d, the immediate early fermentation phase is suitable for adding *W*. *paramesenteroides* and *T*. *halophilus*, which are associated with amino acid metabolisms; the early and middle fermentation phases are appropriate for adding *L*. *plantarum* and *P*. *pentosaceus*, linked to organic acids metabolisms, and adding *W. paramesenteroides* and *T. halophilus* that are associated with amino acid metabolisms; the late phase may incorporate *T. halophilus, P. pentosaceus, C*. *orthopsilosis,* and *Z*. *rouxii*, which are associated with the production of aldehydes, ketones, and esters. Notably, lactic acid synthesis experienced three distinct phases (0–5 d, 5–10 d, and 10–20 d) in the process of soy sauce fermentation (Figure 4c), indicating the addition of different LAB at three different times.

Functions of some of these core microorganisms and their corresponding activation phases in the process of soy sauce fermentation have been investigated in previous studies. *W. paramesenteroides, L. plantarum*, and *P. pentosaceus* exhibited potential capabilities in acid production and enhancing glutamic acid content. Consequently, studies have explored fortifying these strains during the koji-making stage or early fermentation phase to improve soy sauce quality [34,35]. *T. halophilus* is often fortified during the early to mid-fermentation stages due to its salt tolerance and potential capability in increasing flavor compounds content in soy sauce [35]. *C. orthopsilosis* and *Z. rouxii* are often used in the late stages of fermentation to produce flavor compounds, due to their preference for acidic environments [36].

### 3.4. Soy Sauce Fermentation with Synthetic Microbial Community

**Figure 6:**
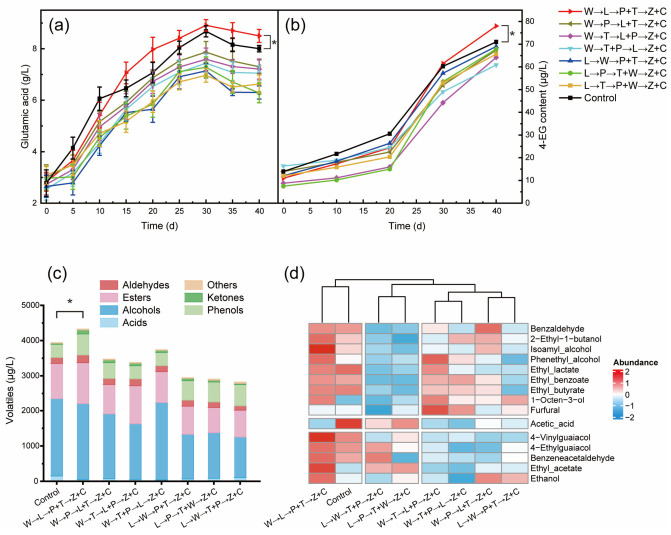
Fermentation of soy sauce with synthetic microbial community: Changes of (**a**) glutamic acid, (**b**) 4-EG contents during soy sauce fermentation; (**c**) Composition of volatiles in soy sauce; (**d**) Clustering analysis of main volatiles in soy sauce (*p* < 0.01). →: Add at different times; +: Add simultaneously. *: significantly greater than control (*p* < 0.05).

Based on the identified core microbes, soy sauce fermentation conducted with different synthetic communities was performed to optimize the assembly of SynCom. These SynComs were designed in accordance with the dominant roles and optimal fermentation periods identified in Section 3.3. The synthetic microbial community combinations were categorized into two major groups: those initiated with *W*. *paramesenteroides* or *L*. *plantarum*, followed by adding other LAB in early and middle phases. Both groups employed *A*. *oryzae* to make the koji and added yeasts in late fermentation phase (Table 1). As shown in Figure 6, in the process of soy sauce fermentation, glutamate levels in both SynCom group W→L→P+T→Z+C and the control group were higher than those of other groups. Interestingly, glutamate and 4-EG contents in group W→L→P+T→Z+C exceeded those of the control group from day 15 and day 30, respectively (Figure 6a,b). After 40 days of fermentation, glutamic acid content in SynCom group W→L→P+T→Z+C reached 8.5 g/L, which was 1.06 times higher than that of the control group. Concurrently, the 4-EG content and total volatiles reached 71.32 μg/L (Figure 6b) and 2855.44 μg/L (Figure 6c) in SynCom group W→L→P+T→Z+C, respectively, representing increases of 1.11 times and 1.25 times compared to the control group.

In terms of volatiles, SynCom group W→L→P+T→Z+C exhibited significantly higher levels of alcohols, esters, and phenolic compounds than those in other SynCom groups (Figure 6c). Furthermore, this group displayed a similar volatile profile to the control group. All volatile compounds except for acetic acid, furfural, and esters in SynCom group W→L→P+T→Z+C were much higher than those in the control group (Figure 6d). This indicated the potential roles of synthetic microbial community in better performance of improving quality and flavor of soy sauce. Preliminary sensory analysis also showed that SynCom group W→L→P+T→Z+C scored higher in umami, sweetness, and smoky taste than the control group (Figure S3).

## 4. Conclusions

This study identified seven core functional microorganisms, including filamentous fungi, yeast, and LAB, involved in soy sauce fermentation through multi-dimensional analysis. Based on metabolic characteristics of the core microorganisms and the features of fermentation process, a four-phase (immediate early, early, middle, late) temporal microbial inoculation strategy was developed for soy sauce fermentation. With the employment of glutamic acid and 4-ethylguaiacol as fermentation indicators, soy sauce fermented by temporal regulation of a synthetic microbial community (SynCom) constructed with the core microorganisms achieved consistent improvement in synthesis of 4-EG, esters, and phenols, compared to traditional fermented soy sauce. Nevertheless, results obtained from this study may have some limitations when applied in industrial soy sauce fermentation. The correlation-guided selection of core microbes may have missed non-dominant but functionally important taxa, as rare species with strong metabolic activities are not well captured by global abundance or network centrality metrics. Moreover, soy sauce fermentations in this work were performed under homogeneous lab conditions. Industrial production of soy sauce moromi systems features oxygen gradients, uneven salt distribution, and different aeration regimes, which likely alter SynCom behaviors. The temporal inoculation strategy may require optimization before transfer to industrial-scale fermentation. Thus, our results should be considered a laboratory-scale proof-of-concept, and pilot-scale validation is required before practical application.

In summary, this study employed multi-omics analytical approaches to elucidate the core microbial consortium and its collaborative role in promotion of formation of umami amino acids and volatiles, providing insights for optimizing soy sauce fermentation process. The exploration of SynComs presents considerable potential for enhancing the sensory quality and functional characteristics of fermented foods, offering both a theoretical foundation and practical guidance for manufacturing soy sauce production.

## Figures and Tables

**Table 1:** Assemble of microbial consortium for construction of synthetic microbiota for soy sauce fermentation.

Groups	Day 0	Day 5	Day 10
*W* *→* *L* *→* *P+T* *→* *Z+C*	*+W. paramesenteroides*	*+L. plantarum*	*+P. pentosaceus* *+T. halophilus*
*W* *→* *P* *→* *L+T* *→* *Z+C*	*+W. paramesenteroides*	*+P. pentosaceus*	*+T. halophilus* *+L. plantarum*
*W* *→* *T* *→* *L+P* *→* *Z+C*	*+W. paramesenteroides*	*+T. halophilus*	*+P. pentosaceus* *+L. plantarum*
*W* *→* *T+P* *→* *L* *→* *Z+C*	*+W. paramesenteroides*	*+T. halophilus* *+P. pentosaceus*	*+L. plantarum*
*L* *→* *W* *→* *P+T* *→* *Z+C*	*+L. plantarum*	*+W. paramesenteroides*	*+T. halophilus* *+P. pentosaceus*
*L* *→* *W* *→* *T+P* *→* *Z+C*	*+L. plantarum*	*+W. paramesenteroides*	*+P. pentosaceus* *+T. halophilus*
*L* *→* *P* *→* *T+W* *→* *Z+C*	*+L. plantarum*	*+T. halophilus*	*+W. paramesenteroides* *+P. pentosaceus*

→, different time intervals; +, addition of corresponding microorganism; Z+C, addition of *Z. rouxii* and *C. orthopsilosis* on day 25.

## Data Availability

The original contributions presented in this study are included in the article/Supplementary Material. Further inquiries can be directed to the corresponding author.

## Supplementary Materials

The following supporting information can be downloaded at: https://www.mdpi.com/article/10.3390/foods15101736/s1. Table S1: Standard curves of main flavor volatiles in soy sauce. Table S2: Dominant microbes in soy sauce fermentation process. Table S3: Correlation center microbes in soy sauce fermentation process. Table S4: Microorganisms related to flavor volatiles in soy sauce. Table S5: Flavor volatiles identified by GC–MS in soy sauce, Table S6: Metabolomics analysis data. Figure S1: Composition of free amino acids during soy sauce fermentation, Figure S2: Microbial compositions during core microbial validation fermentation process. Figure S3: Sensory evaluation of SynCom fermented soy sauce. Figure S4: Microbial diversity index statistics and rarefaction analysis.

## References

[1] Devanthi P.V.P., Gkatzionis K. (2019). Soy sauce fermentation: Microorganisms, aroma formation, and process modification. Food Res. Int..

[2] Chen C., Wen L.F., Yang L.X., Li J., Kan Q.X., Xu T., Liu Z., Fu J.Y., Cao Y. (2023). Metagenomic and metaproteomic analyses of microbial amino acid metabolism during Cantonese soy sauce fermentation. Front. Nutr..

[3] Diez-Simon C., Eichelsheim C., Mumm R., Hall R.D. (2020). Chemical and sensory characteristics of soy sauce: A Review. J. Agric. Food Chem..

[4] Yang Y., Deng Y., Jin Y.L., Liu Y.X., Xia B.X., Sun Q. (2017). Dynamics of microbial community during the extremely long-term fermentation process of a traditional soy sauce. J. Sci. Food Agric..

[5] Tian W., Zhao S., Wang Q., Wang W., He J., Dong B., Zhao G. (2025). Influence of spatial and temporal diversity and succession of microbial communities on physicochemical properties and flavor substances of soy sauce. Food Chem..

[6] Feng Y.Z., Xie Z.M., Huang M.T., Tong X., Hou S., Tin H., Zhao M.M. (2024). Decoding temperature-driven microbial community changes and flavor regulation mechanism during winter fermentation of soy sauce. Food Res. Int..

[7] Pan F.S., Qiu S.Y., Lv Y.Y., Li D.A. (2023). Exploring the controllability of the Baijiu fermentation process with microbiota orientation. Food Res. Int..

[8] Jia Y., Niu C.T., Lu Z.M., Zhang X.J., Chai L.J., Shi J.S., Xu Z.H., Li Q. (2020). A bottom-up approach to develop a synthetic microbial community model: Application for efficient reduced-salt broad bean paste fermentation. Appl. Environ. Microbiol..

[9] Nguyen N.T.H., Huang M.B., Liu F.Y., Huang W.L., Tran H.T., Hsu T.W., Huang C.L., Chiang T.Y. (2023). Deciphering microbial community dynamics along the fermentation course of soy sauce under different temperatures using metagenomic analysis. Biosci. Microb. Food Health.

[10] Song Z.W., Du H., Zhang Y., Xu Y. (2017). Unraveling core functional microbiota in traditional solid-state fermentation by high-throughput amplicons and metatranscriptomics sequencing. Front. Microbiol..

[11] Tan G.L., Hu M., Li X.L., Li X.Y., Pan Z.Q., Li M., Li L., Wang Y., Zheng Z.Y. (2022). Microbial community and metabolite dynamics during soy sauce koji making. Front. Microbiol..

[12] Liu H., Zhou W., Lu J., Wu D., Ge F. (2025). Construction of a synthetic microbial community and its application in salt-reduced soy sauce fermentation. Food Microbiol..

[13] Peng Q., Quan L., Zheng H., Li J., Xie G. (2025). Analyzing the contribution of top-down and bottom-up methods to the construction of synthetic microbial communities in Jiuyao. Food Microbiol..

[14] Cui Y.H., Qu X.J., Li H.M., He S.H., Liang H.Y., Zhang H., Ma Y. (2012). Isolation of halophilic lactic acid bacteria from traditional Chinese fermented soybean paste and assessment of the isolates for industrial potential. Eur. Food Res. Technol..

[15] Hu G., Chen J., Du G., Fang F. (2023). Moromi mash dysbiosis trigged by salt reduction is relevant to quality and aroma changes of soy sauce. Food Chem..

[16] Chen C., Hou S., Wu C., Cao Y., Tong X., Chen Y. (2023). Improving protein utilization and fermentation quality of soy sauce by adding protease. J. Food Compos. Anal..

[17] Zhang L., Zhang Z., Huang J., Zhou R.Q., Wu C.D. (2024). Revealing salt concentration for microbial balance and metabolite enrichment in secondary fortified fermented soy sauce: A multi-omics perspective. Food Chem. X.

[18] Ma Z.Y., Zhang X.M., Wang R., Wang M., Liu T., Tan Z.L. (2020). Effects of Chemical and mechanical lysis on microbial DNA yield, integrity, and downstream amplicon sequencing of rumen bacteria and protozoa. Front. Microbiol..

[19] Gao L., Zhou J., He G.Q. (2022). Effect of microbial interaction on flavor quality in Chinese baijiu fermentation. Front. Nutr..

[20] Liu H., Chen X.G., Lu J., Wu D.H. (2024). Evaluation of the differences between low-salt solid-state fermented soy sauce and high-salt diluted-state fermented soy sauce in China: From taste-active compounds and aroma-active compounds to sensory characteristics. J. Sci. Food Agric..

[21] (2001). Fermented Soy Sauce.

[22] Hu Q., Cheng S.Q., Qian D.S., Wang Y.Y., Xie G.F., Peng Q. (2025). Identification of core microbial communities and their influence on flavor-oriented traditional fermented sour cucumbers. Food Microbiol..

[23] Zhang L., Xiong S., Du T., Xu Y., Zhao X., Huang G., Guan Q., Xiong T. (2024). Unraveling the core functional microbiota involved in metabolic network of characteristic flavor development during soy sauce fermentation. Food Biosci..

[24] Zhao C., Lin J., Zhang Y., Wu H., Li W., Lin W., Luo L. (2024). Comprehensive analysis of flavor formation mechanisms in the mechanized preparation Cantonese soy sauce koji using absolute quantitative metabolomics and microbiomics approaches. Food Res. Int..

[25] Zhang L., Xiong S., Du T., Xiao M., Peng Z., Xie M., Guan Q., Xiong T. (2023). Effects of microbial succession on the dynamics of flavor metabolites and physicochemical properties during soy sauce koji making. Food Biosci..

[26] Zhang L., Zhang Y., Huang J., Zhou R., Wu C. (2026). A synthetic microbial community enhances flavor and safety in reduced-salt soy sauce fermentation: Multi-omics insights into microbial stabilization and metabolic regulation. Food Microbiol..

[27] Li S.Y., Guo L.J., Gu J.H., Mu G.Q., Tuo Y. (2023). Screening lactic acid bacteria and yeast strains for soybean paste fermentation in northeast of China. Food Sci. Nutr..

[28] Li W.W., Zhang H., Wang R.N., Zhang C.N., Li X.T. (2024). Temporal profile of the microbial community and volatile compounds in the third-round fermentation of sauce-flavor baijiu in the Beijing region. Foods.

[29] Liu Y.H., Sun G.R., Li J.Y., Cheng P., Song Q., Lv W., Wang C.L. (2024). Starter molds and multi-enzyme catalysis in koji fermentation of soy sauce brewing: A review. Food Res. Int..

[30] Wei Q.Z., Wang H.B., Lv Z.J., Hu G., Li Y., Liu Y.H., Wang Y., Lu F.P. (2013). Search for potential molecular indices for the fermentation progress of soy sauce through dynamic changes of volatile compounds. Food Res. Int..

[31] Wang S., Wu Q., Nie Y., Wu J., Xu Y. (2019). Construction of synthetic microbiota for reproducible flavor compound metabolism in Chinese light-aroma-type liquor produced by solid-state fermentation. Appl. Environ. Microbiol..

[32] Xie J., Hong J., Zhang C., Yuan X., Zhao Z., Zhao D., Wang S., Sun B., Ao R., Sun J. (2025). Machine learning-assisted identification of core flavor compounds and prediction of core microorganisms in fermentation grains and pit mud during the fermentation process of strong-flavor Baijiu. Food Chem..

[33] Huang X.X., Lin H.Y., Wang Z.Y., Zhao M.M., Feng Y.Z. (2025). Optimization of derivatization-gas chromatography/mass spectrometry (Der-GC/MS) for analyzing non-volatile metabolites in soy sauce koji-making process and their evolution patterns. Food Chem..

[34] Hu G., Wang Y., Chen J., Du G., Fang F. (2024). Synergistic fermentation with functional bacteria for production of salt-reduced soy sauce with enhanced aroma and saltiness. Food Biosci..

[35] Wu S., Sun Z., Guo J., Li D., Lin X., Zhang S., Ji C. (2025). Improving soy sauce quality via co-fermentation with indigenous *Tetragenococcus*: Reducing biogenic amines and enhancing flavor. Food Res. Int..

[36] Kuang X., Su H., Li W., Lin L., Lin W., Luo L. (2022). Effects of microbial community structure and its co-occurrence on the dynamic changes of physicochemical properties and free amino acids in the Cantonese soy sauce fermentation process. Food Res. Int..

