# Supplementary material for: Construction of Synthetic Microbial Community with Core Microorganisms for Soy Sauce Fermentation
Source: Foods. 2026 May 14;15(10):1736. doi: 10.3390/foods15101736 (PMC13206497; doi:10.3390/foods15101736)

1. Bacterial Alpha Diversity Index Statistics

Sample	Number	OTUs	Shannon	Chao	Ace	Simpson	Shannoneven	Coverage
0	42726.0	67.0	1.70	72.0	74.45	0.30	0.40	1.00
5	37091.0	67.0	1.81	70.46	74.96	0.23	0.43	1.00
10	36269.0	70.0	1.80	78.75	82.18	0.24	0.42	1.00
15	77073.0	62.0	1.67	66.67	65.31	0.26	0.41	1.00
20	33610.0	68.0	1.94	74.6	75.52	0.19	0.46	1.00
25	44817.0	73.0	1.82	78.5	81.01	0.23	0.42	1.00
30	35447.0	58.0	1.38	61.11	63.37	0.41	0.34	1.00
35	33013.0	66.0	1.80	81.11	81.79	0.23	0.43	1.00
40	36065.0	64.0	1.62	71.33	71.65	0.28	0.39	1.00
55	36479.0	68.0	1.73	74.0	79.98	0.29	0.41	1.00

2. Bacterial Alpha Diversity rarefaction analysis

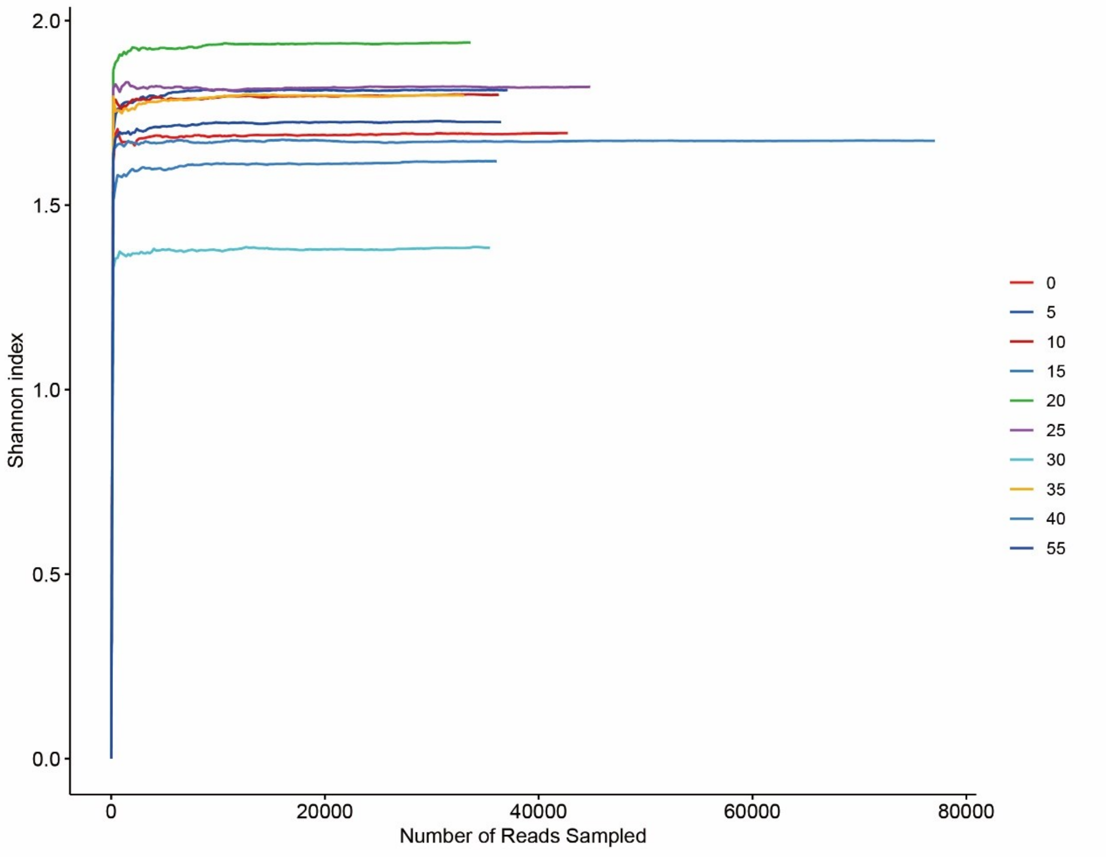

3. Fungal Alpha Diversity Index Statistics

Sample	Number	OTUs	Shannon	Chao	Ace	Simpson	Shannoneven	Coverage
0	44656.0	18.0	0.14	25.5	24.43	0.95	0.05	1.00
5	43468.0	19.0	0.14	20.0	24.56	0.95	0.05	1.00
10	57664.0	20.0	0.14	20.75	21.97	0.95	0.05	1.00
15	44622.0	18.0	0.14	18.75	19.94	0.95	0.05	1.00
20	44697.0	23.0	0.14	30.0	38.92	0.95	0.05	1.00
25	107423.0	33.0	0.14	45.0	51.30	0.95	0.04	1.00
30	79707.0	32.0	0.33	35.33	34.41	0.88	0.09	1.00
35	91136.0	33.0	0.34	34.0	35.05	0.86	0.10	1.00
40	110262.0	34.0	0.34	36.5	38.99	0.86	0.10	1.00
55	40433.0	23.0	0.31	28.25	34.61	0.89	0.10	1.00

4. Fungal Alpha Diversity rarefaction analysis

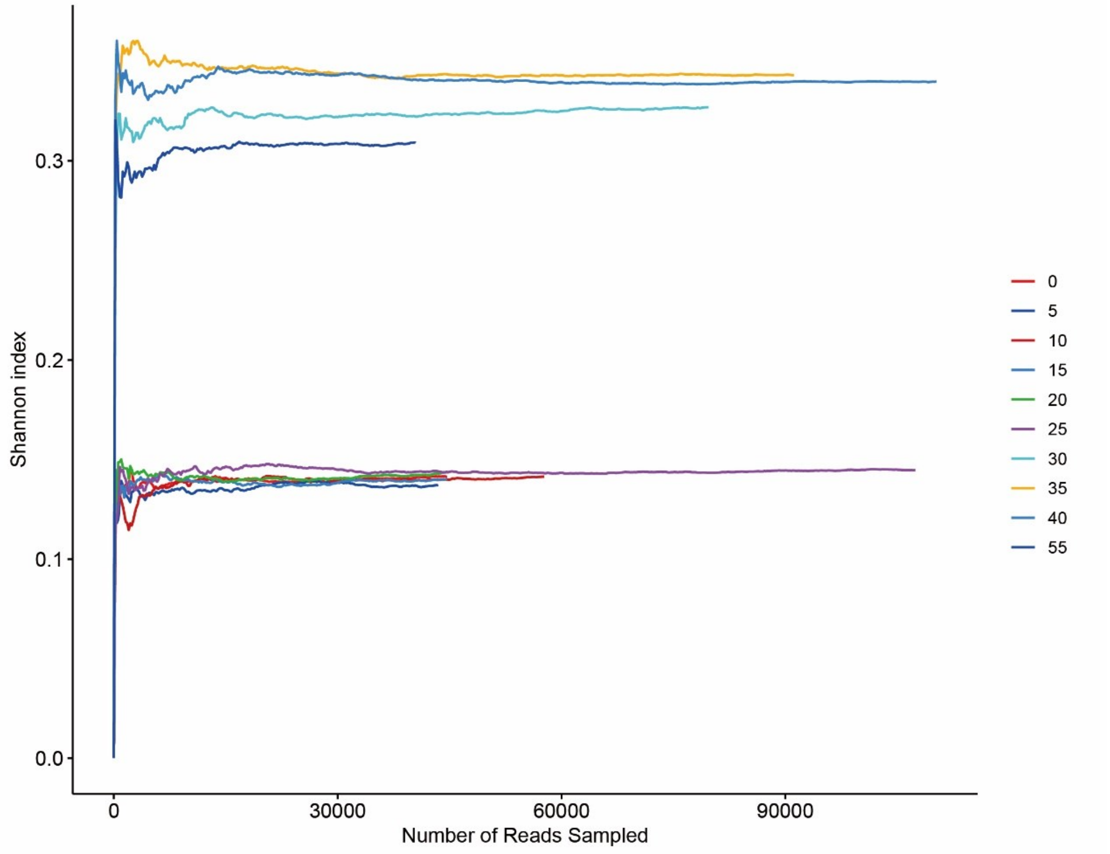

Supplement: Supplementary file 1 [file foods-15-01736-s001.zip › Figure S4.pdf]
