# Supplementary material for: Construction of Synthetic Microbial Community with Core Microorganisms for Soy Sauce Fermentation
Source: Foods. 2026 May 14;15(10):1736. doi: 10.3390/foods15101736 (PMC13206497; doi:10.3390/foods15101736)
Supplement: Supplementary file 1 [file foods-15-01736-s001.zip › Table S1.pdf]

Table S1 Standard curves of main flavor volatiles in soy sauce

Compounds	Retention time (s)	Standard curves	R^2
4-Vinylguaiacol	1213.31	y = 0.1268x + 0.0832	0.994
4-Ethylguaiacol	1116.73	y = 0.1615x + 0.0153	0.950
Benzaldehyde	778.697	y = 2.1334x + 0.1323	0.960
Isoamyl acetate	413.796	y = 0.985x + 0.052	0.998
Ethyl acetate	199.48	y = 0.432x + 0.102	0.974
Ethyl benzoate	972.56	y = 0.892x + 0.043	0.999
Phenethyl alcohol	1043.14	y = 0.691x + 0.047	0.965
Ethanol	229.018	y = 0.289x + 0.082	0.981
Isoamyl alcohol	496.637	y = 0.764x + 0.039	0.985
Lactic acid	719.296	y = 0.127x + 0.033	0.957
