# Supplementary material for: Construction of Synthetic Microbial Community with Core Microorganisms for Soy Sauce Fermentation
Source: Foods. 2026 May 14;15(10):1736. doi: 10.3390/foods15101736 (PMC13206497; doi:10.3390/foods15101736)
Supplement: Supplementary file 1 [file foods-15-01736-s001.zip › Table S2.pdf]

Table S2 Dominant microbes in soy sauce fermentation process

	Microbial species	Average relative abundance	Taxonomy
1	*Weissella paramesenteroides*	31.3%	*Bacteria*
2	*Lactiplantibacillus plantarum*	18.1%	*Bacteria*
3	*Tetragenococcus halophilus*	15.1%	*Bacteria*
4	*Pediococcus pentosaceus*	7.6%	*Bacteria*
5	*Weissella cibaria*	5.8%	*Bacteria*
6	*Staphylococcus epidermidis*	5.2%	*Bacteria*
7	*Staphylococcus gallinarum*	5.1%	*Bacteria*
8	*Enterobacter hormaechei*	2.9%	*Bacteria*
9	*Pediococcus acidilactici*	2.3%	*Bacteria*
10	*Lactococcus lactis*	2.1%	*Bacteria*
11	*Bacillus velezensis*	2.1%	*Bacteria*
12	*Enterobacter kobei*	1.8%	*Bacteria*
13	*Aspergillus oryzae*	81.2%	*Fungi*
14	*Zygosaccharomyces rouxii*	12.5%	*Fungi*
15	*Candida orthopsilosis*	6.5%	*Fungi*
16	*Millerozyma farinosa*	4.5%	*Fungi*
17	*Pichia membranifaciens*	1.7%	*Fungi*
