# Supplementary material for: Construction of Synthetic Microbial Community with Core Microorganisms for Soy Sauce Fermentation
Source: Foods. 2026 May 14;15(10):1736. doi: 10.3390/foods15101736 (PMC13206497; doi:10.3390/foods15101736)
Supplement: Supplementary file 1 [file foods-15-01736-s001.zip › Table S3.pdf]

Table S3 Correlation center microbes in soy sauce fermentation process

	Microbial species	Number of collinear lines	Average relative abundance	Taxonomy
1	*Candida orthopsilosis*	14	6.5%	*Fungi*
2	*Weissella paramesenteroides*	14	31.3%	*Bacteria*
3	*Zygosaccharomyces rouxii*	13	12.5%	*Fungi*
4	*Tetragenococcus halophilus*	13	15.1%	*Bacteria*
5	*Lactiplantibacillus plantarum*	13	18.1%	*Bacteria*
6	*Pichia membranifaciens*	12	1.7%	*Fungi*
7	*Pediococcus acidilactici*	12	2.3%	*Bacteria*
8	*Aspergillus oryzae*	11	81.2%	*Fungi*
9	*Meyerozyma guilliermondii*	11	2.1%	*Fungi*
10	*Weissella cibaria*	11	5.8%	*Bacteria*
11	*Millerozyma farinosa*	10	4.5%	*Fungi*
12	*Candida psychrophila*	10	1.8%	*Fungi*
13	*Staphylococcus epidermidis*	10	5.2%	*Bacteria*
14	*Lactococcus lactis*	9	2.1%	*Bacteria*
15	*Lactobacillus fermentum*	9	0.8%	*Bacteria*
16	*Pediococcus pentosaceus*	9	7.6%	*Bacteria*
