# Supplementary material for: Construction of Synthetic Microbial Community with Core Microorganisms for Soy Sauce Fermentation
Source: Foods. 2026 May 14;15(10):1736. doi: 10.3390/foods15101736 (PMC13206497; doi:10.3390/foods15101736)
Supplement: Supplementary file 1 [file foods-15-01736-s001.zip › Table S4.pdf]

Table S4 Microorganisms related to mainly flavor volatiles in soy sauce

	Microbial species	Number of related flavor volatiles	Average relative abundance	Taxonomy
1	*Weissella paramesenteroides*	9	31.3%	*Bacteria*
2	*Lactiplantibacillus plantarum*	8	18.1%	*Bacteria*
3	*Lactobacillus fermentans*	8	0.8%	*Bacteria*
4	*Saccharopolyspora thermophilu*	6	2.1%	*Bacteria*
5	*Tetragenococcus halophilus*	5	15.1%	*Bacteria*
6	*Pediococcus pentosaceus*	9	7.6%	*Bacteria*
7	*Pediococcus acidilactici*	6	2.3%	*Bacteria*
8	*Staphylococcus epidermidis*	6	5.2%	*Bacteria*
9	*Bacillus velezensis*	5	2.1%	*Bacteria*
10	*Zygosaccharomyces rouxii*	15	12.5%	*Fungi*
11	*Pichia fermentans*	14	0.7%	*Fungi*
12	*Candida orthopsilosis*	16	6.5%	*Fungi*
13	*Meyerozyma guilliermondii*	15	0.8%	*Fungi*
14	*Aspergillus oryzae*	5	81.2%	*Fungi*
15	*Candida guilliermondii*	8	0.7%	*Fungi*
