# Supplementary material for: Construction of Synthetic Microbial Community with Core Microorganisms for Soy Sauce Fermentation
Source: Foods. 2026 May 14;15(10):1736. doi: 10.3390/foods15101736 (PMC13206497; doi:10.3390/foods15101736)
Supplement: Supplementary file 1 [file foods-15-01736-s001.zip › Table S5.pdf]

Name	R.T. (s)[a]	CAS	Content/(μg/L)							
			Control	W→L→P+T	W→P→L+T	W→T→L+P	W→T+P→L	L→W→P+T	L→P→T+W	L→T→P+W
			**Alcohols**							
1-Butanol, 2-ethyl-	589.493	97-95-0	65.15	78.39	69.16	53.16	68.15	55.95	42.66	31.53
1-Butanol	436.402	71-36-3	0.38	0.52	-[b]	-	0.42	-	-	7.18
1-Butanol, 3-methyl-	496.637	123-51-3	108.91	149.21	115.16	96.16	91.63	89.65	80.49	68.86
1-Dodecanol	1069.96	112-53-8	2.37	0.81	1.20	1.09	-	-	0.56	0.79
1-Heptanol	713.648	111-70-6	-	2.53	2.94	6.11	14.23	3.50	0.81	-
1-Hexanol	628.867	111-27-3	4.86	0.26	1.65	0.15	0.03	0.17	0.11	109.88
1-Hexanol, 2-ethyl-	741.055	104-76-7	0.11	1.19	1.10	1.03	0.57	2.11	0.34	0.45
1-Nonanol	867.623	143-08-8	-	-	-	-	-	60.63	-	24.25
1-Octanol	792.887	111-87-5	0.87	1.43	1.51	1.41	2.53	1.95	0.49	6.14
1-Octen-3-ol	709.8	3391-86-4	19.44	31.49	26.31	31.46	26.45	29.66	18.46	22.16
1-Propanol, 2-methyl-	382.088	78-83-1	92.03	26.55	17.62	6.10	3.47	10.16	5.81	0.94
1-Propanol, 3-(methylthio)-	912.755	505-10-2	13.11	12.49	1.73	1.07	0.47	1.44	1.23	0.60

2,3-Butanediol	807.049	513-85-9	1.88	47.72	8.18	2.85	10.51	-	19.81	60.71
2-Butanol, 2,3-dimethyl-	392.289	594-60-5	0.30	1.46	-	-	-	-	-	-
2-Furanmethanol	871.857	98-00-0	24.02	30.84	8.58	10.94	1.83	3.83	6.24	19.03
2-Octen-1-ol, (E)-	836.892	18409-17-1	0.66	0.75	0.89	-	2.72	39.37	0.31	-
2-Nonanol	763.039	628-99-9	1.92	-	1.81	3.10	8.65	3.99	0.36	-
2-Propen-1-ol	406.459	107-18-6	0.15	-	-	-	-	-	-	0.06
3-Buten-1-ol, 3-methyl-	537.449	763-32-6	0.54	2.42	1.38	0.56	0.42	1.02	0.81	-
3-Hexanol, 2-methyl-	643.12	617-29-8	2.33	0.12	-	-	-	-	-	-
3-Octen-2-ol, (Z)-	718.734	69668-89-9	0.50	15.75	15.97	11.47	1.02	-	4.27	-
3-Octanol	663.38	589-98-0	1.50	-	-	-	-	0.06	-	-
Benzyl alcohol	1020.67	100-51-6	-	2.28	1.52	1.01	0.40	1.92	0.65	1.81
Cyclodecanol	602.132	2566-44-1	0.05	22.13	0.38	0.18	-	-	-	-
Ethanol	229.018	64-17-5	421.36	653.15	609.51	377.93	174.37	547.46	332.37	463.91
Ethanol, 2-phenoxy-	1182.03	122-99-6	-	0.08	0.26	0.12	0.01	0.06	0.03	-

Phenylethyl Alcohol	1043.14	60-12-8	133.93	177.53	124.66	80.16	141.66	86.13	100.46	110.68
Subtotal			896.37	1259.11	1011.51	686.06	549.54	939.07	616.26	928.99
**Phenols**										
2-Methoxy-4-vinylphenol	1213.31	7786-61-0	130.82	233.64	95.13	112.62	122.65	102.35	111.63	150.16
Eugenol	1196.15	97-53-0	-	0.49	0.16	0.13	0.15	0.18	0.22	4.34
Maltol	1079.01	118-71-8	40.73	1.55	0.14	0.11	0.10	0.23	0.96	3.47
Phenol	1102.16	108-95-2	7.50	0.90	0.77	0.45	0.28	1.99	0.37	3.99
Mequinol	1320.52	150-76-5	6.06	-	-	-	-	0.13	-	0.06
Phenol, 2,6-dimethoxy-	1249.65	91-10-1	0.05	0.43	-	0.07	0.08	-	0.14	-
Phenol, 2-methoxy-	1011.93	90-05-1	0.96	4.32	3.16	3.74	1.37	41.67	1.55	0.69
Phenol, 2-methyl-	1099.47	95-48-7	0.27	0.02	-	-	-	-	-	-
Phenol, 4-ethyl-	1199.91	123-07-9	-	0.08	0.60	-	0.12	1.12	0.15	17.93
Phenol, 4-ethyl-2-methoxy-	1116.73	2785-89-9	68.47	71.32	51.41	58.42	49.88	60.88	67.85	61.66
Subtotal			254.87	312.75	151.37	175.54	174.62	208.55	182.88	242.30
**Pyrazines**										
Pyrazine	504.766	290-37-9	0.30	1.56	1.55	0.42	0.18	1.37	-	-
Pyrazine, 2,3-dimethyl-	627.614	5910-89-4	7.99	0.95	0.31	0.56	0.11	0.77	0.47	-
Pyrazine, 2,5-dimethyl-	605.628	123-32-	0.14	3.46	31.34	1.60	4.19	42.64	1.34	0.07

		0								
Pyrazine, 2,6-dimethyl-	610.945	108-50-9	0.31	14.85	8.55	2.20	0.87	5.33	1.18	2.86
Pyrazine, ethenyl-	706.159	4177-16-6	-	0.14	0.27	0.20	-	0.07	-	-
Pyrazine, methyl-	555.34	109-08-0	1.47	3.42	4.57	2.38	0.60	1.89	0.58	0.02
Pyrazine, tetramethyl-	732.238	1124-11-4	-	1.75	0.11	0.38	-	0.37	0.09	12.64
Pyrazine, trimethyl-	675.543	14667-55-1	1.02	1.85	6.69	1.41	1.60	6.81	1.31	0.72
Pyridine, 2,4,6-trimethyl-	701.188	108-75-8	11.97	8.57	-	-	-	-	-	-
Subtotal			23.22	36.54	53.40	9.16	7.54	59.25	4.97	16.30
					**Aldehydes**					
2-Hexenal, 2-methyl-	474.855	28467-88-1	1.78	2.78	3.76	1.62	0.01	2.53	-	0.10
2-Methylbutyraldehyde	564.32	96-17-3	0.45	1.33	0.03	0.01	0.10	30.15	-	-
2-Pentenal, 2-methyl-	456.356	623-36-9	0.29	0.02	0.22	0.30	0.03	0.61	-	-
5-Methyl-2-phenyl-2-hexenal	1144.7	21834-92-4	1.49	8.06	1.70	1.50	2.26	2.60	1.76	0.13
Benzaldehyde	778.697	100-52-7	53.41	54.74	59.16	43.16	32.16	34.16	21.16	25.13
Benzeneacetaldehyde	867.179	122-78-1	100.67	113.21	66.13	77.16	71.26	84.16	109.16	52.49

Benzeneacetaldehyde, α-ethylidene-	1059.66	4411-89-6	2.76	0.40	0.17	2.43	1.96	16.16	-	1.29
Butanal, 3-methyl-	212.512	590-86-3	0.98	76.34	44.65	20.67	9.22	37.29	48.80	-
Decanal	753.744	112-31-2	-	0.03	3.20	1.02	1.09	2.45	1.45	-
Dodecanal	908.342	112-54-9	-	0.54	0.25	0.14	0.22	0.31	0.18	-
Furfural	729.559	98-01-1	51.99	51.46	48.16	77.16	67.49	55.94	26.67	49.72
Hexanal	526.32	66-25-1	-	2.12	-	-	-	0.20	-	-
Methional	722.711	3268-49-3	4.91	20.70	8.88	6.99	3.04	5.25	10.74	15.65
Nonanal	669.11	124-19-6	1.33	3.61	2.82	1.15	0.54	2.35	1.33	7.53
Octanal	601.23	124-13-0	-	-	-	-	-	3.35	-	-
Pentadecanal-	1111.96	2765-11-9	-	-	-	0.02	-	0.02	-	0.03
Tetradecanal	1047.35	124-25-4	-	0.47	0.32	-	0.19	0.31	-	-
Benzaldehyde, 2-ethyl-	935.464	22927-13-5	-	0.15	0.22	-	0.27	-	-	-
Subtotal			220.05	335.97	239.66	233.33	189.86	277.83	221.25	152.07
					**Acids**					
Acetic acid	719.296	64-19-7	128.38	48.93	73.21	52.48	38.49	64.79	84.17	104.46
Benzoic acid	1339.72	65-85-0	1.14	1.02	0.38	0.29	0.20	0.41	47.01	15.55

Butanoic acid	850.835	107-92-6	-	0.46	-	-	-	-	0.29	22.43
Butanoic acid, 2-methyl-	880.194	116-53-0	21.29	42.71	31.94	11.03	-	-	11.53	11.73
Dodecanoic acid	1358.89	143-07-7	-	1.91	0.66	0.51	0.46	1.23	0.91	43.06
Heptanoic acid	1064.54	111-14-8	2.56	0.83	0.68	0.48	0.22	-	0.35	27.40
Hexanoic acid	997.572	142-62-1	2.60	1.21	1.76	0.73	0.14	57.72	0.27	235.64
Linoelaidic acid	1275.28	506-21-8	1.40	0.97	0.87	-	-	0.40	-	-
n-Decanoic acid	1247.6	334-48-5	2.45	3.25	0.72	0.65	0.55	1.10	1.06	18.89
Nonanoic acid	1189.4	112-05-0	81.18	22.17	4.15	3.69	3.59	5.47	5.64	54.94
Octanoic acid	1128.52	124-07-2	0.19	2.12	1.16	0.63	0.36	0.84	0.56	57.40
Pentanoic acid	926.914	109-52-4	2.38	0.01	0.08	-	-	-	-	17.36
Pentanoic acid, 4-methyl-	970.366	646-07-1	0.11	1.55	0.52	0.57	0.12	0.59	0.48	1.71
Propanedioic acid, phenyl-	1411.66	2613-89-0	0.17	0.69	-	-	-	0.26	0.28	14.36
Propanoic acid	785.519	79-09-4	2.37	3.09	1.53	1.54	0.64	67.15	52.04	-
Propanoic acid, 2-	806.597	79-31-2	-	47.30	-	7.29	-	-	13.96	-

methyl-										
Tetradecanoic acid	1501.65	544-63-8	-	0.68	0.32	0.21	0.14	0.31	0.27	9.65
Subtotal			246.20	178.89	117.98	80.12	44.93	200.26	218.79	634.57
**Ketones**										
1-Octen-3-one	876.196	4312-99-6	0.19	22.33	-	-	-	-	-	-
2-Butanone	321.23	78-93-3	-	0.09	-	0.04	0.01	61.96	0.04	0.01
2-Heptanone	476.871	110-43-0	0.14	0.41	1.13	0.17	0.72	0.60	0.56	0.15
2-Hexanone, 5-methyl-	436.14	110-12-3	2.10	-	2.10	0.69	0.06	4.49	0.63	0.03
2-Octanone	573.847	111-13-7	2.42	45.54	30.24	21.59	34.66	29.73	13.58	4.62
2-Pentanone	345.32	107-87-9	1.72	1.55	0.32	0.07	0.03	1.47	0.15	0.09
3(2H)-Furanone, 4-hydroxy-5-methyl-	1165.57	19322-27-1	12.01	6.56	1.66	1.97	0.46	1.11	1.65	1.88
3-Octanone	545.874	106-68-3	0.66	1.97	6.70	0.20	4.40	4.52	1.45	-
7-Octen-2-one	1061.01	3664-60-6	0.38	1.81	0.23	0.29	0.31	0.29	0.20	0.91
Acetone	163.131	67-64-1	0.05	1.34	89.03	45.77	34.39	76.27	72.48	21.18
Benzylacetone	986.21	2550-26-7	30.01	30.47	0.32	0.09	0.22	1.02	0.90	-
Furaneol	1114.99	3658-	10.45	15.51	0.21	0.36	0.06	0.02	0.30	-

		77-3								
Methyl furaneol	642.32	27538-09-6	41.21	40.20	0.25	0.83	0.13	1.10	0.17	-
Subtotal			101.16	145.36	132.18	72.03	75.42	120.63	92.06	28.87
					**Esters**					
Diisobutyl phthalate	1399.73	84-69-5	0.13	0.66	0.81	0.55	0.29	1.20	0.51	0.18
1-Butanol, 3-methyl-, acetate	413.796	123-92-2	41.09	42.71	16.59	0.72	-	0.93	0.21	0.41
2-Furanmethanol, acetate	781.994	623-17-6	-	0.78	0.89	0.42	-	-	0.05	4.78
Acetic acid, 2-phenylethyl ester	982.553	103-45-7	2.17	30.54	3.86	21.59	0.90	-	-	8.26
Benzeneacetic acid, ethyl ester	962.315	101-97-3	7.38	25.15	5.40	1.89	0.88	10.51	2.30	25.58
Benzenepropanoic acid, ethyl ester	1027.53	2021-28-5	-	9.16	0.92	-	0.01	0.02	0.05	25.49
Ethyl butyrate	645.25	123-86-4	77.37	76.69	61.23	66.13	64.95	31.68	39.16	30.16
Decanoic acid, ethyl ester	854.92	110-38-3	0.12	4.31	3.03	0.17	-	0.26	13.50	32.32
Dodecanoic acid, ethyl ester	994.714	106-33-2	0.93	1.59	0.19	3.10	0.46	0.20	0.53	2.57
Ethyl Acetate	199.48	141-78-6	70.92	121.45	78.65	64.21	55.16	76.51	98.16	103.16
Ethyl benzoate	972.56	93-89-0	60.68	64.16	51.36	59.36	61.45	34.32	25.12	24.58
Ethyl lactate	1118.13	97-64-3	94.60	91.39	65.37	89.88	70.84	64.95	54.85	59.82

Heptanoic acid, ethyl ester	615.52	106-30-9	0.36	3.10	0.54	0.21	0.01	-	-	49.09
Hexadecanoic acid, ethyl ester	1237.68	628-97-7	62.23	51.30	0.65	0.49	0.46	2.19	1.81	64.45
Hexanoic acid, ethyl ester	523.957	123-66-0	3.07	0.15	0.66	0.24	0.06	0.10	0.05	14.69
Hexanoic acid, hexyl ester	835.037	6378-65-0	-	4.69	1.36	-	0.05	-	-	23.06
Isopentyl hexanoate	719.851	2198-61-0	-	6.89	0.38	0.11	1.95	0.57	2.43	29.65
Linoleic acid ethyl ester	1386.23	544-35-4	81.82	10.20	0.90	0.46	0.44	1.21	0.62	16.75
Methyl acetate	416.32	79-20-9	-	3.98	1.16	0.91	0.17	-	4.13	-
Butanoic acid, 2-methyl-, ethyl ester	345.741	7452-79-1	7.09	10.02	4.10	0.07	0.51	-	0.01	-
Butanoic acid, 3-methyl-, ethyl ester	359.339	108-64-5	-	2.15	3.78	0.08	0.24	6.86	1.69	-
Subtotal			509.97	561.07	301.83	310.60	258.85	231.51	245.18	515.01
					**Others**					
1-Dodecene	532.773	112-41-4	0.75	-	1.44	-	0.28	-	-	0.01
2-Acetylthiazole	868.961	24295-03-2	20.20	1.59	12.97	-	0.02	-	-	1.22
Benzenamine, 4-methoxy-	1125.67	104-94-9	-	-	-	-	-	-	-	0.08
Benzonitrile	841.198	100-47-	0.11	2.22	2.57	0.22	0.37	3.35	0.38	0.47

		0								
Benzyl nitrile	1058.55	140-29-4	0.02	5.18	3.59	2.43	-	-	2.19	0.43
Butanenitrile, 3-methyl-	422.716	625-28-5	15.04	13.50	9.89	2.44	1.22	5.29	4.08	0.09
Formamide	957.854	75-12-7	0.43	-	1.52	-	0.67	0.13	0.98	1.44
Formic acid hydrazide	879.797	624-84-0	0.48	-	31.94	11.03	0.40	34.20	11.53	-
Succinic anhydride	497.074	108-30-5	0.31	-	73.74	13.49	0.19	-	0.05	0.30
Benzofuran	763.679	271-89-6	0.42	0.42	3.64	8.65	3.99	0.19	0.51	0.36
Benzofuran, 2,3-dihydro-	1318.3	496-16-2	0.37	0.37	0.21	0.31	0.35	0.32	0.57	-
Furan, 2-pentyl-	524.117	3777-69-3	0.05	0.05	0.11	0.01	0.10	0.07	0.53	0.39
Furan, 3-phenyl-	1009.77	13679-41-9	2.42	2.42	2.76	1.52	1.68	2.35	3.64	1.42
Subtotal			40.59	25.75	144.39	40.10	9.27	45.90	24.47	6.20

[a]: flavor compounds retention time; [b]:Not detect
