# Supplementary figures and images for: Construction of Synthetic Microbial Community with Core Microorganisms for Soy Sauce Fermentation
Source: Foods. 2026 May 14;15(10):1736. doi: 10.3390/foods15101736 (PMC13206497; doi:10.3390/foods15101736)

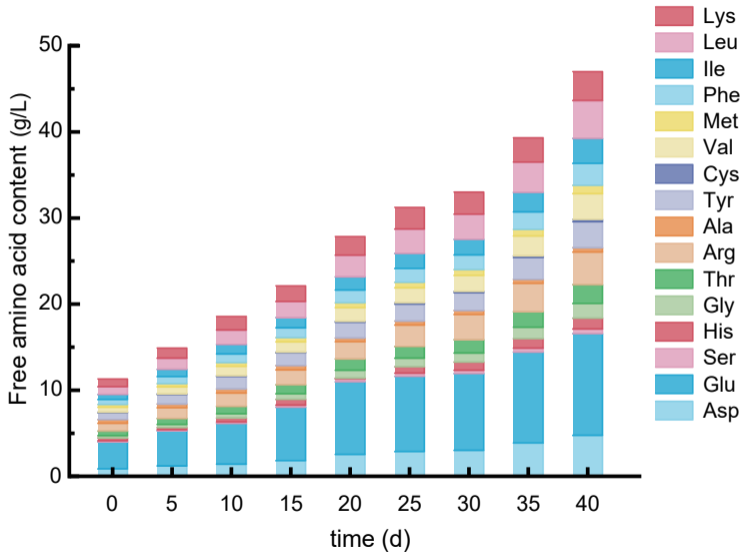

Supplement: Supplementary file 1 [file foods-15-01736-s001.zip › Figure S1.pdf]

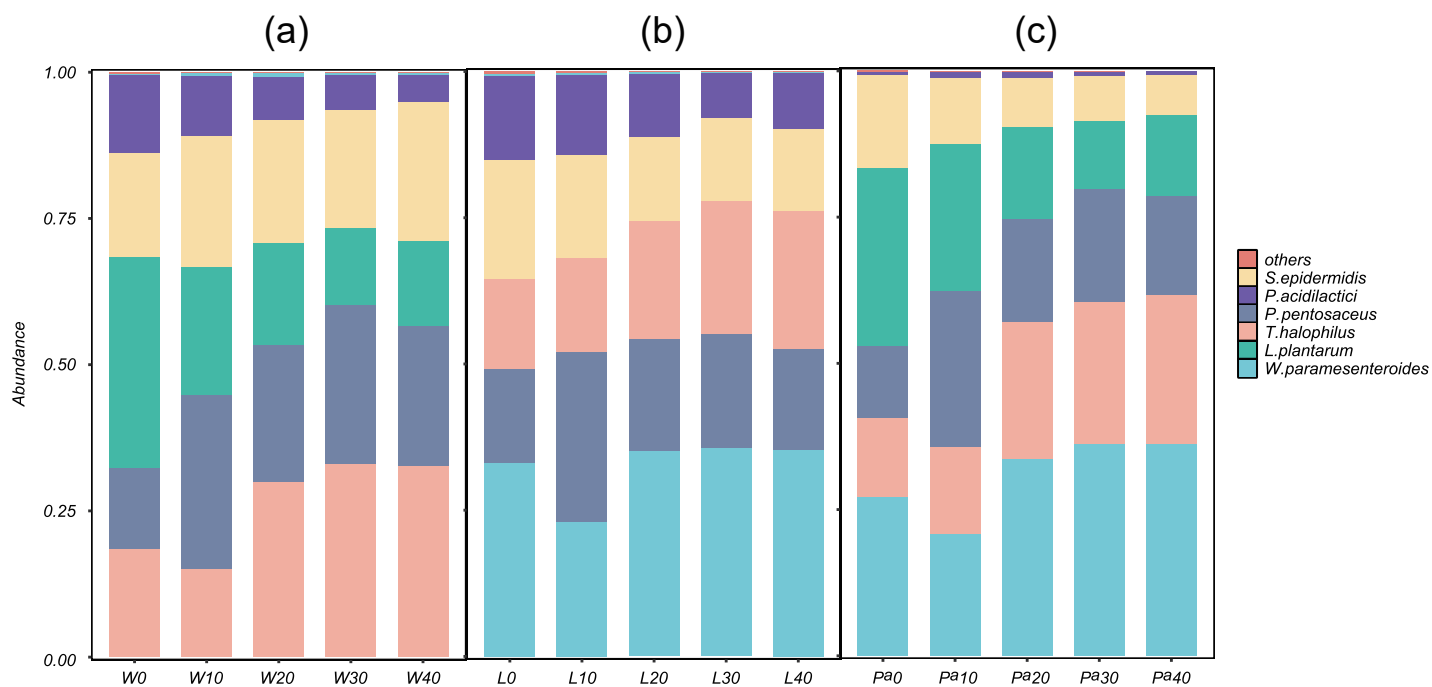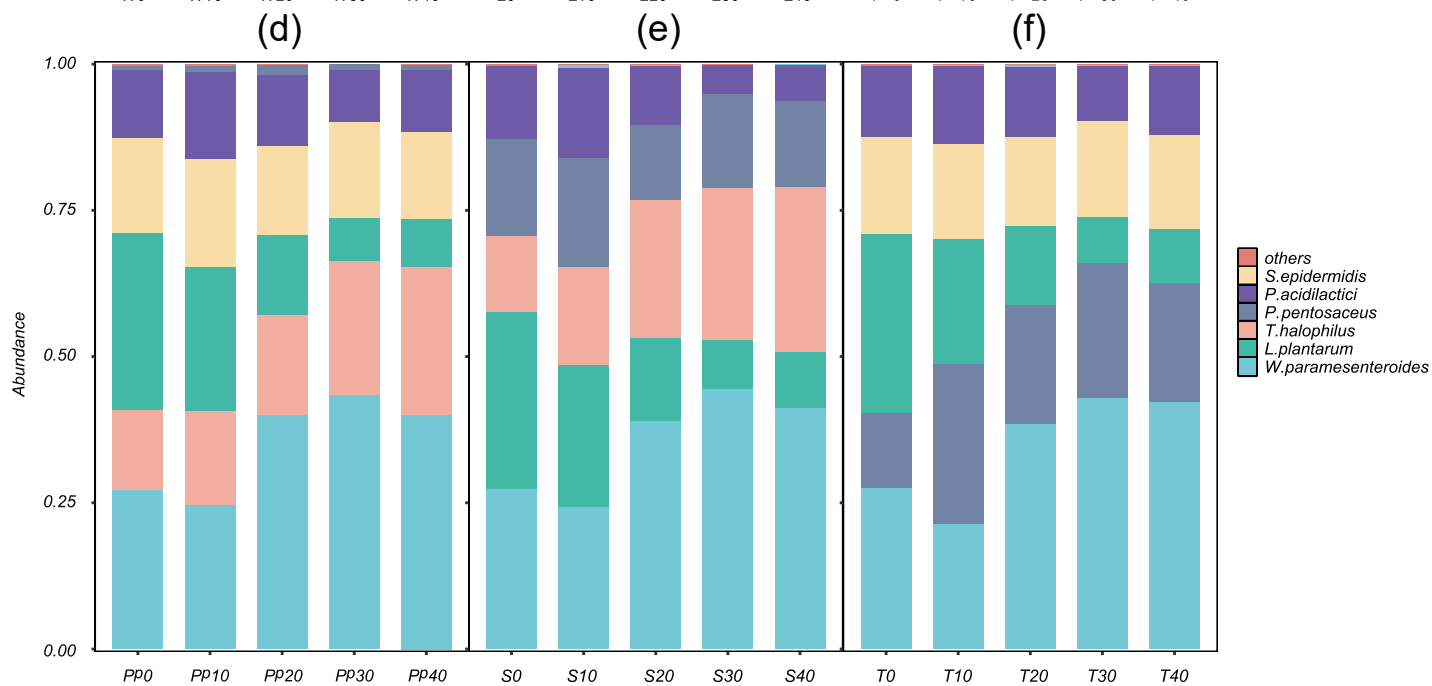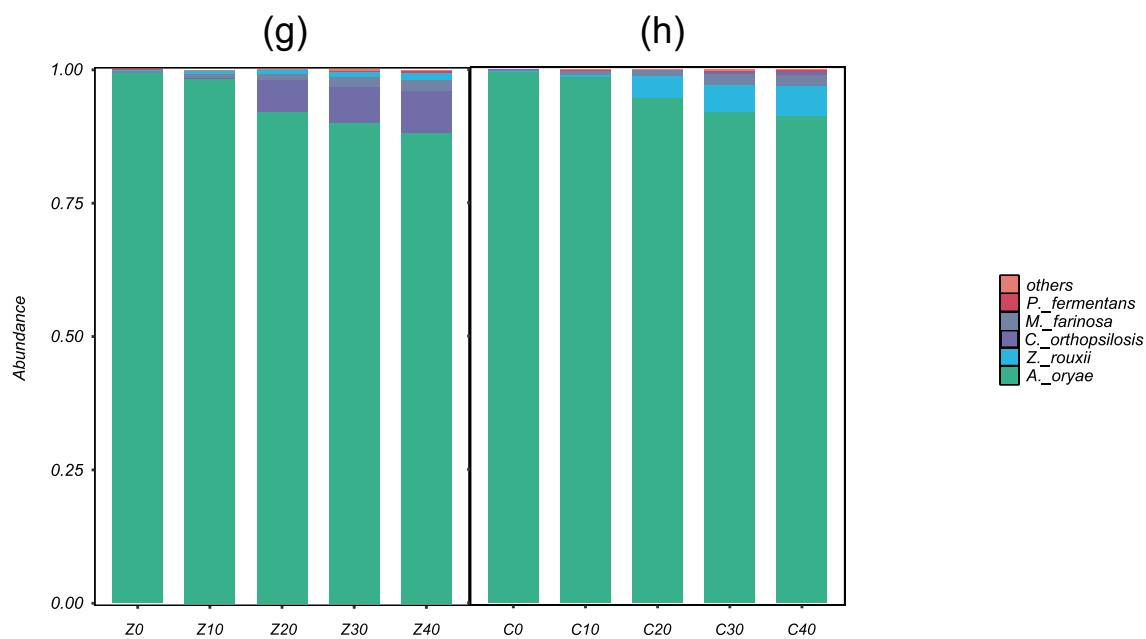

Supplement: Supplementary file 1 [file foods-15-01736-s001.zip › Figure S2.pdf]

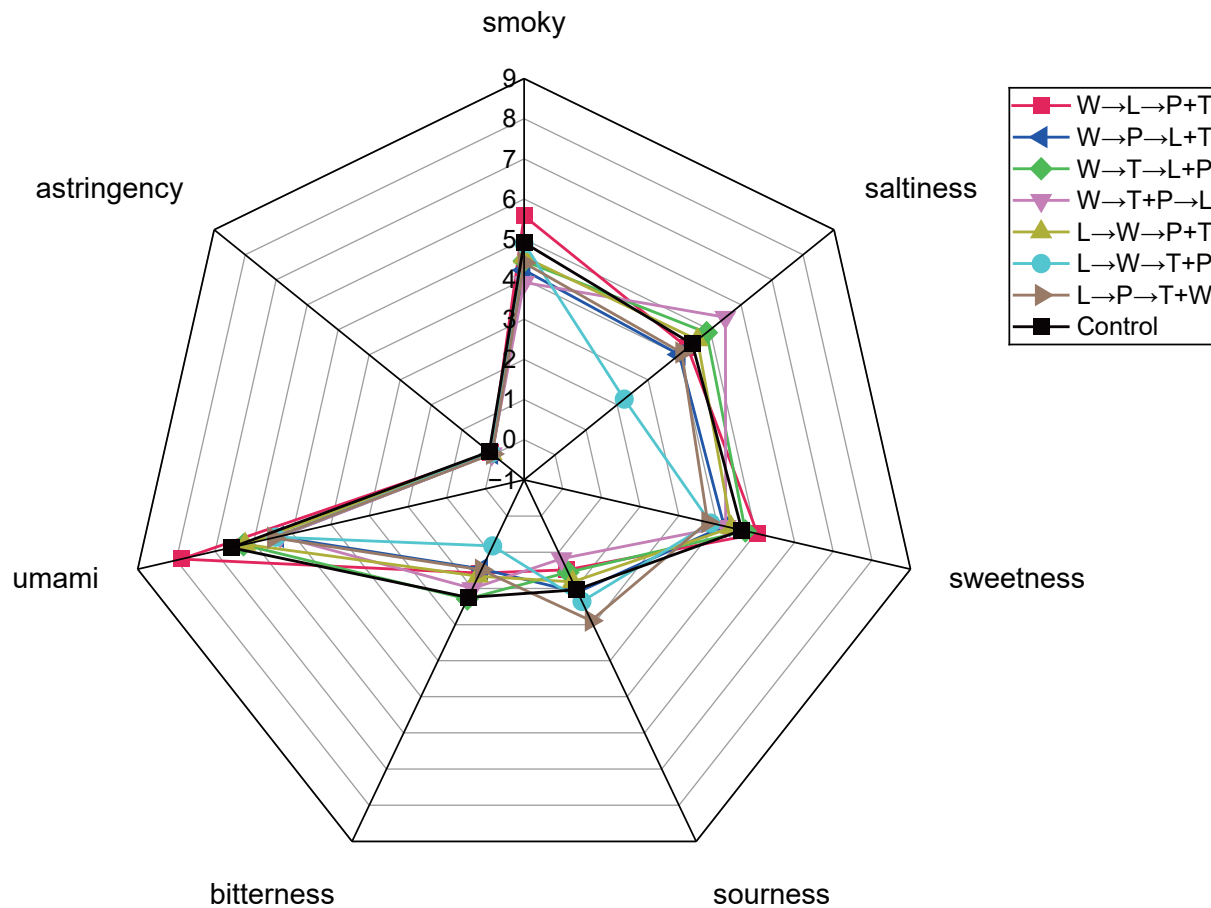

Supplement: Supplementary file 1 [file foods-15-01736-s001.zip › Figure S3.pdf]
